# Supplementary material for: Structures of the ApoL1 and ApoL2 N-terminal domains reveal a non-classical four-helix bundle motif
Source: Commun Biol. 2021 Jul 27;4:916. doi: 10.1038/s42003-021-02387-5 (PMC8316464; doi:10.1038/s42003-021-02387-5)
Supplement: Supplementary file 2 — Supplementary Information [file 42003_2021_2387_MOESM2_ESM.pdf]

Supplementary Information for

Structures of the ApoL1 and ApoL2 N-terminal domains reveal a non-classical four-helix bundle motif

Mark Ultsch[1], Michael J. Holliday[2], Stefan Gerhardy[2], Paul Moran[2], Suzie J. Scales[3], Nidhi Gupta[3], Francesca Oltrabella[3], Cecilia Chiu[4], Wayne Fairbrother[2], Charles Eigenbrot[1], Daniel Kirchhofer[2*]

From the Departments of [1]Structural Biology, [2]Early Discovery Biochemistry, [3]Immunology and [4]Antibody Engineering, Genentech Inc., 1 DNA Way, South San Francisco, CA 94080

This file includes:

Supplementary Table 1
Supplementary Figures 1-9

Supplementary Table 1

Supplementary Table 1. Kinetic constants of ApoL1 binding to antibodies Ab7D6, Ab6D12 and Ab3B6

Antibody	k_{on} (M^{-1} s^{-1})	k_{off} (s^{-1})	K_D (nM)
Ab6D12	$4.8 \pm 0.3 \times 10^5$	$4.1 \pm 0.1 \times 10^{-4}$	0.86 ± 0.20
Ab3B6	$5.9 \pm 0.4 \times 10^4$	$9.2 \pm 0.7 \times 10^{-5}$	1.56 ± 0.04
Ab7D6	$1.8 \pm 0.1 \times 10^5$	$2.9 \pm 0.1 \times 10^{-4}$	1.63 ± 0.10

Anti-ApoL1 antibodies were captured on anti-murine Fc-coated CM5 biosensor chips and the binding of serially diluted ApoL1 was measured by determining the kinetic constants (k_{on}, k_{off}) and calculating the K_D (k_{off}/k_{on}). The values are the average ± S.D. of three independent experiments.

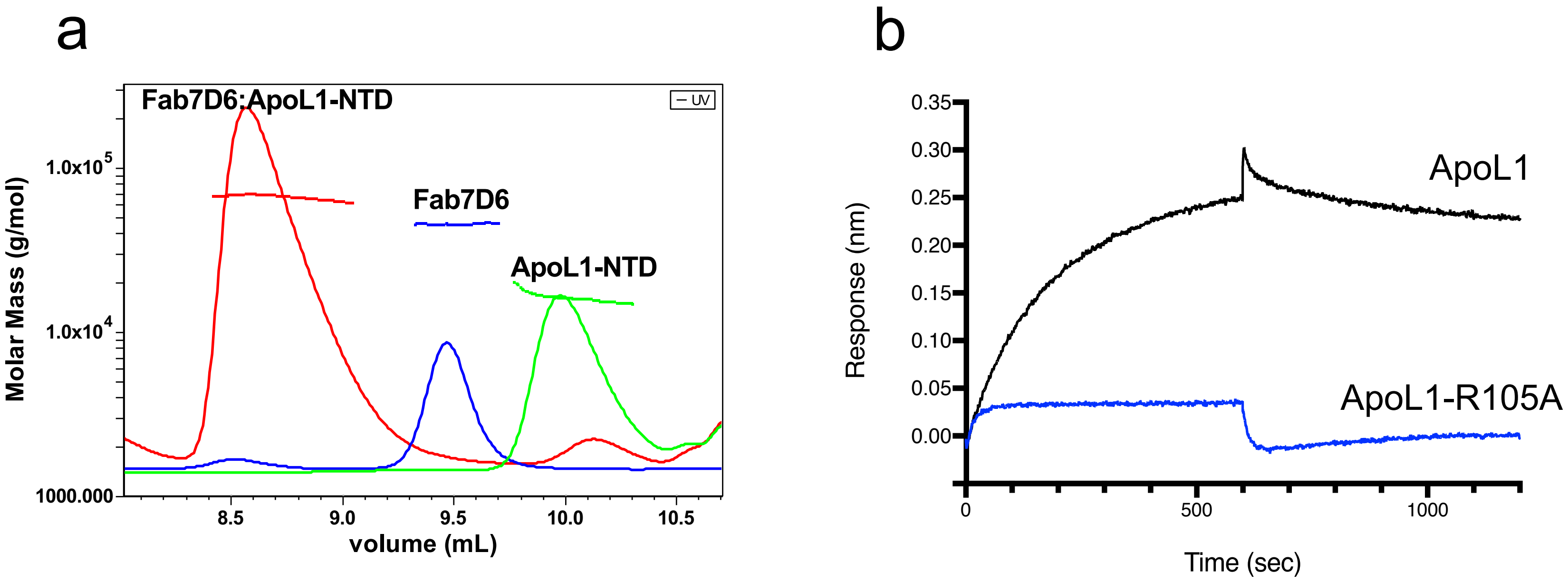

Supplementary Figure 1. Stoichiometry of the Fab7D6:ApoL1-NTD complex and importance of ApoL1-R105 for Fab6D12 binding. **a.** SEC-MALS shows that ApoL1-NTD is a monomer, which forms a 1:1 complex with Fab7D6. The average molecular mass ± S.D. (three independent experiments) of ApoL1-NTD, Fab7D6 and the Fab7D6:ApoL1-NTD complex were 15.3 ± 2.0, 44.8 ± 0.4 and 65.0 ± 0.9 kDa, respectively. **b.** Biolayer interferometry assay with immobilized Ab6D12 showing loss of binding by the ApoL1-R105A mutant in comparison to ApoL1 (both at 50 nM).

a

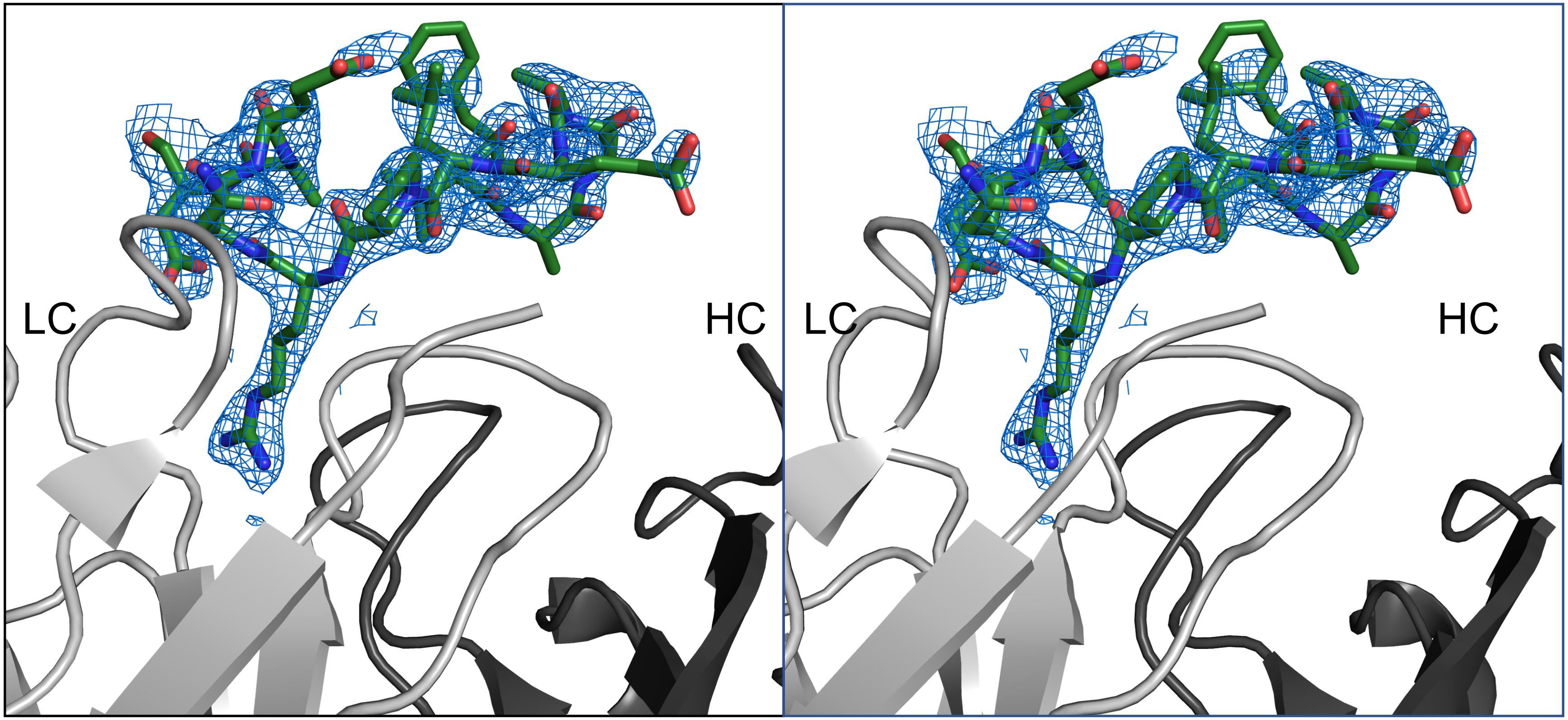

Supplementary Figure 2. Electron density 2mFo-DFc omit maps (in stereo) contoured at 1 times rmsd shown for a subset of atoms of ApoL1- and ApoL2-NTD bound to different Fabs (light chain LC in light grey and heavy chain HC in dark grey). **a.** Fab6D12 and ApoL1-NTD residues 97-109 (dark green).

b

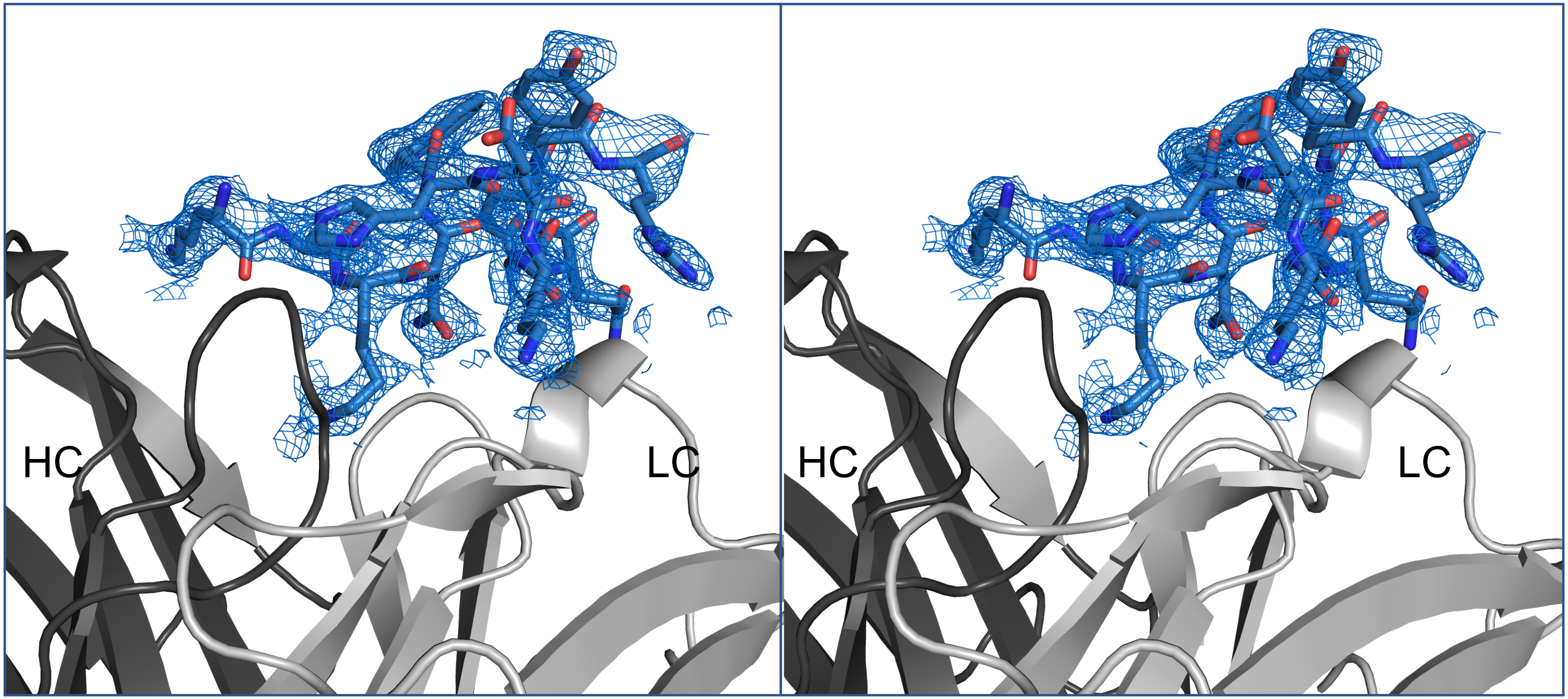

Supplementary Figure 2. b. Fab3B6 and ApoL1-NTD residues 125-137 (blue).

c

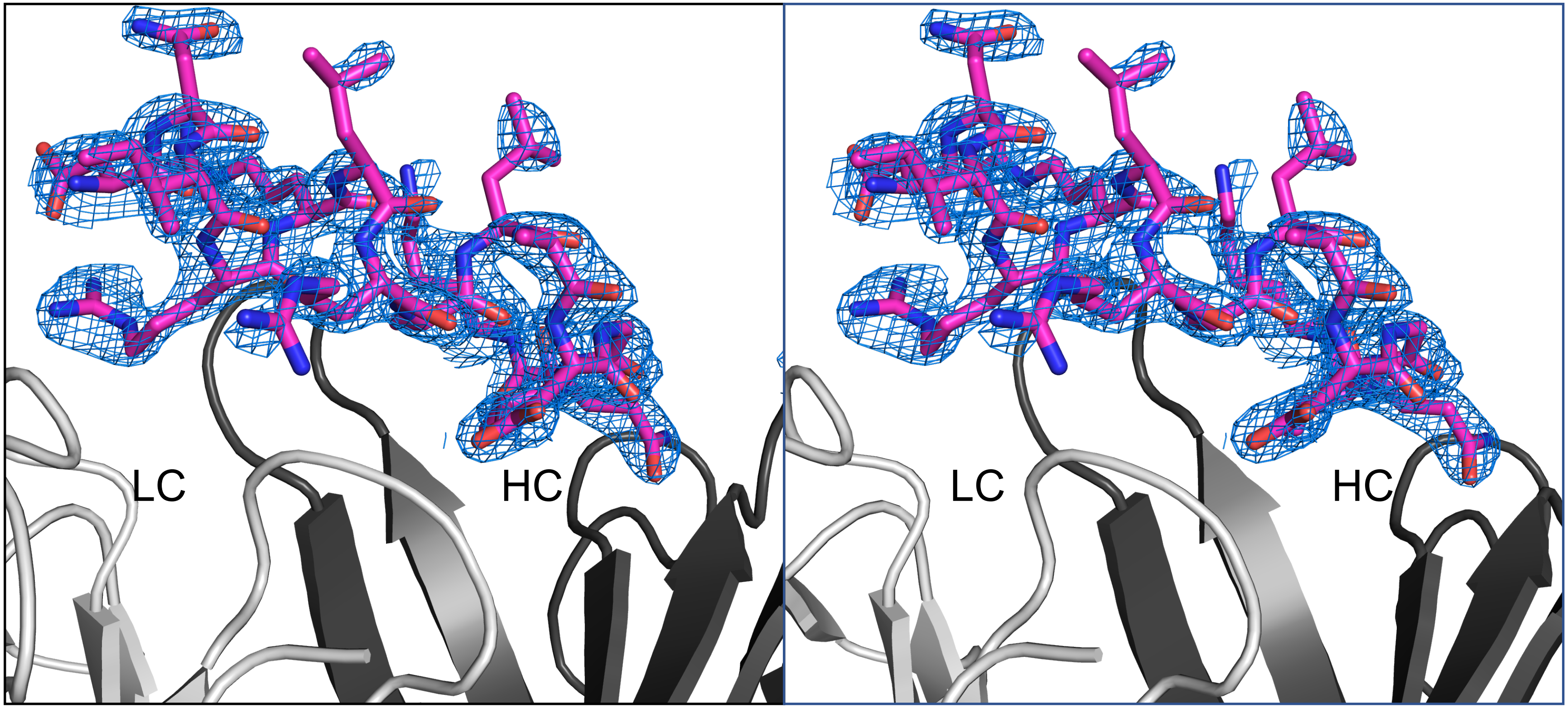

Supplementary Figure 2. c. Fab7D6 and ApoL1-NTD residues 153-167 (magenta) in the domain-swapped dimer.

d

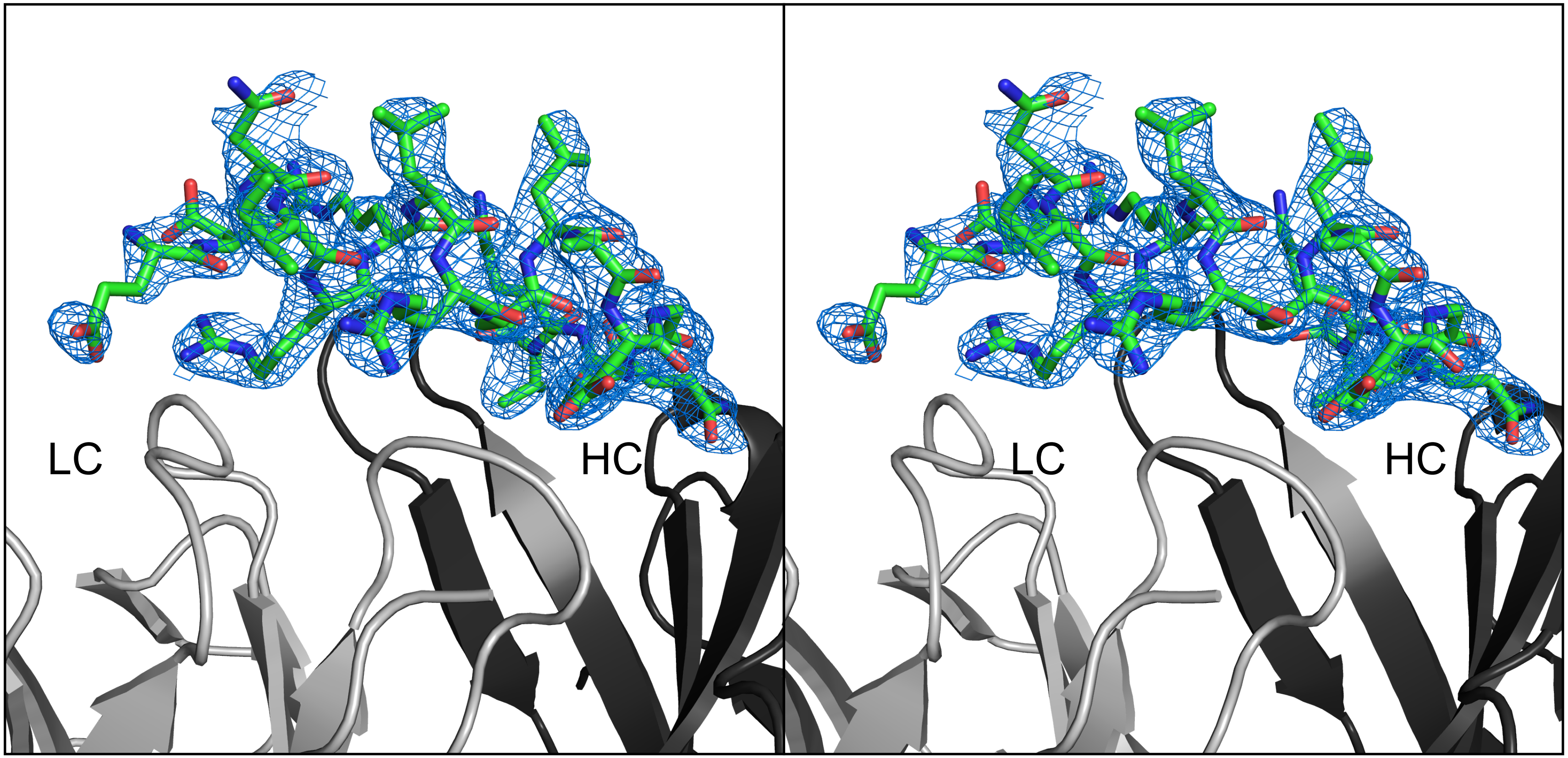

Supplementary Figure 2. d. Fab7D6 and ApoL1-peptide residues 152-168 (green).

e

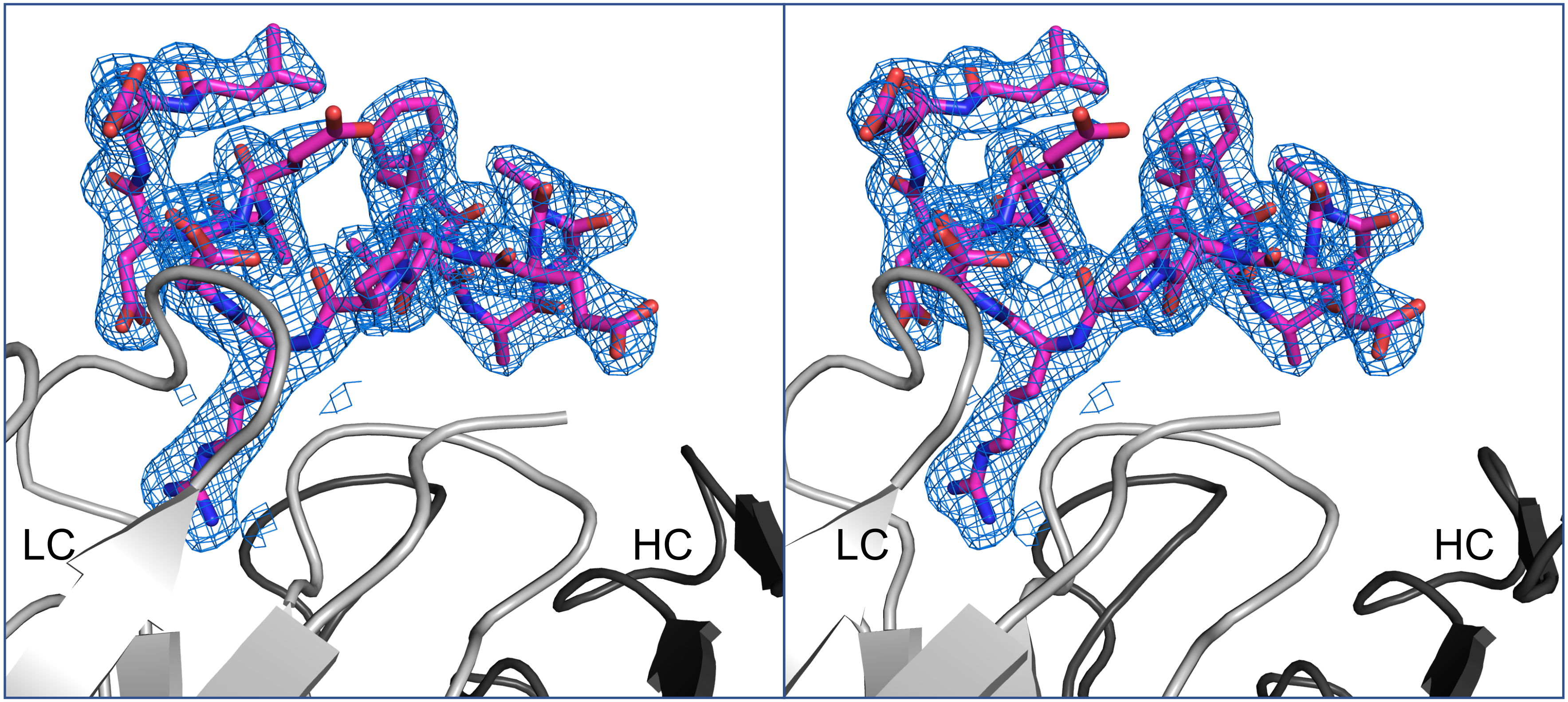

Supplementary Figure 2. e. Fab6D12 and ApoL2-NTD residues 38-52 (magenta).

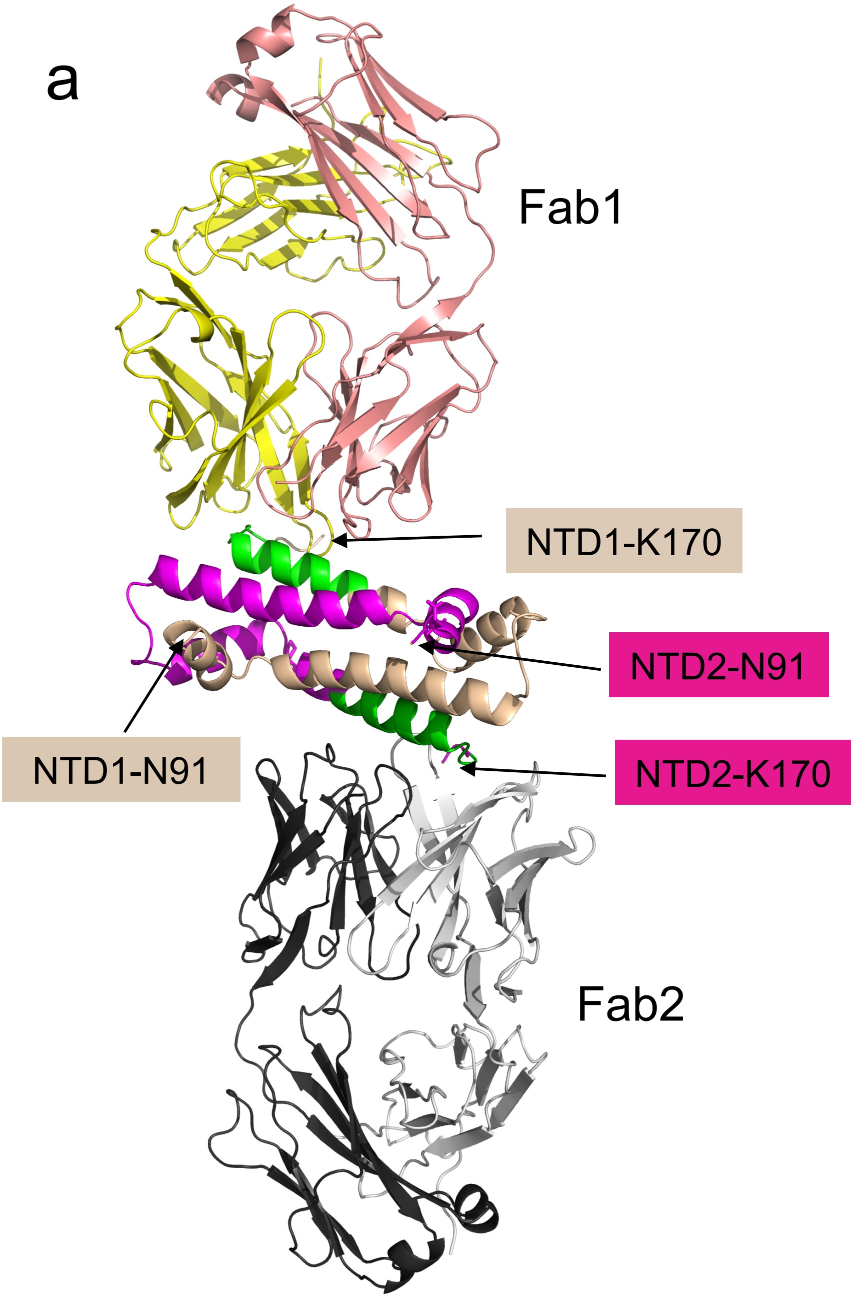

Supplementary Figure 3. The ApoL1-NTD swapped-dimer. **a.** Structure of Fab7D6 bound to ApoL1-NTD in relation to its symmetry mate shows the NTD domain swap. Complex 1 composition: Fab1 (pink light chain; yellow heavy chain) bound to NTD1 (brown, BH3-like region in green). Complex 2 composition (symmetry mate): Fab 2 (light grey heavy chain; dark grey light chain) bound to NTD2 (magenta, green BH3-like region).

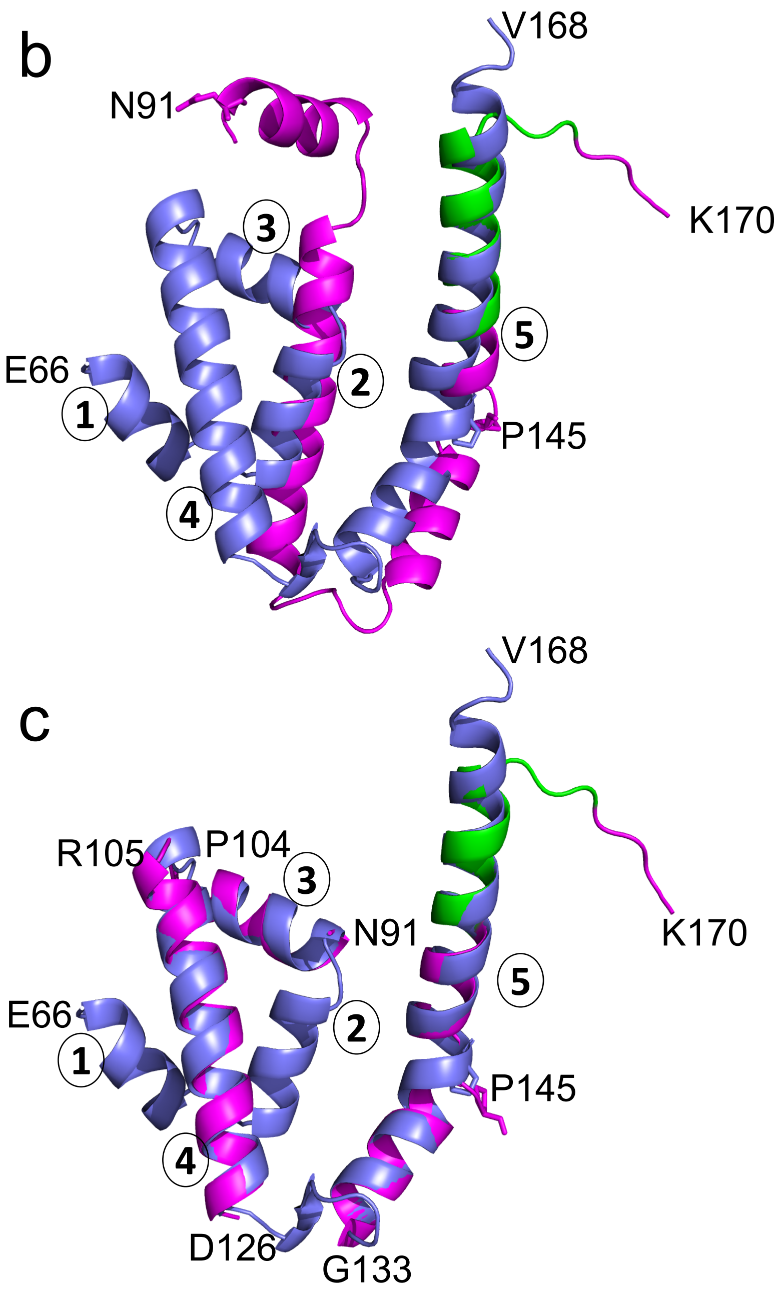

Supplementary Figure 3. b. Superposition of the NTD2 (magenta with green BH3-like region) from the Fab7D6 complex with the NTD from the Fab3B6 complex (blue; helices 1-5 are indicated) (rmsd of 3.2 Å for 172 atoms, residues 134-168). **c.** Selected helical stretches of the NTD2 (magenta with green BH3-like region) from the Fab7D6 complex superimpose well with the NTD of the Fab3B6 complex (blue): residues N91-P104 with rmsd of 0.8 for 73 atoms; residues R105-D126 with rmsd of 0.8 Å for 118 atoms; residues G133-P145 with rmsd of 1.0 for 69 atoms, residues P145-V168 with rmsd of 0.8 for 91 atoms.

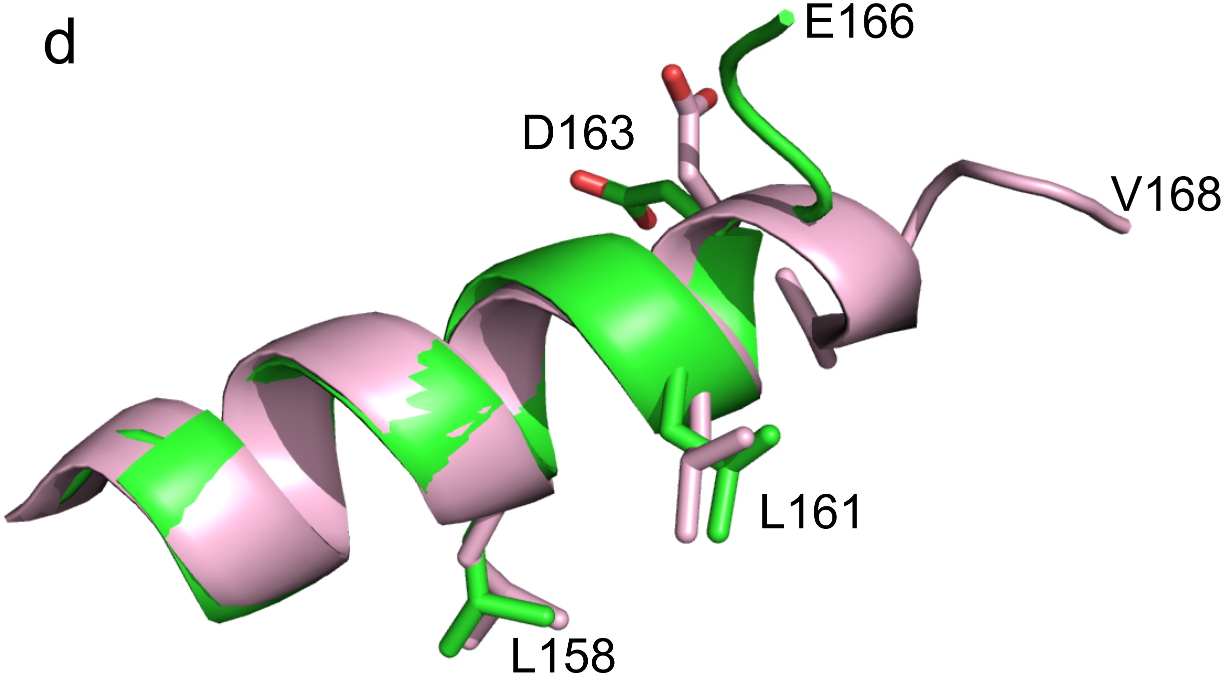

Supplementary Figure 3. d. Superposition of the BH3-like helices from the Fab3B6:ApoL1-NTD complex (pink) and the Fab7D6-peptide complex (green).

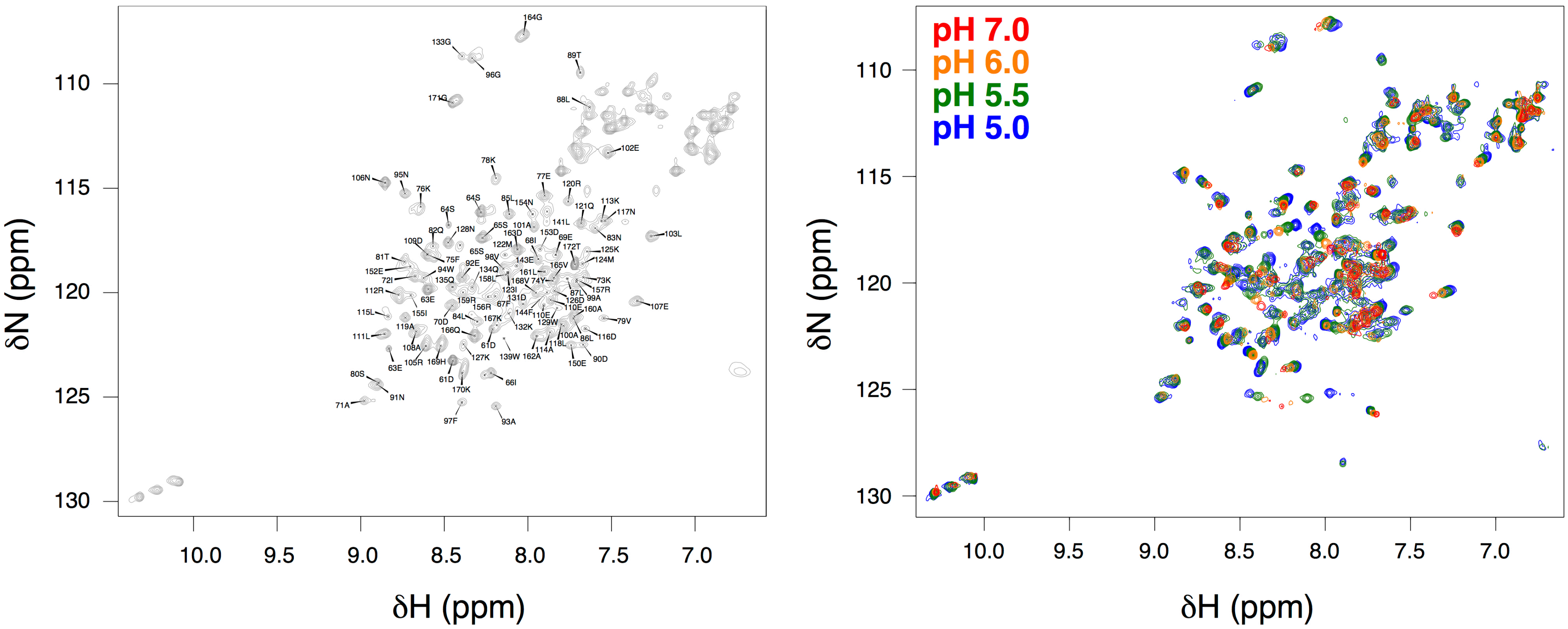

Supplementary Figure 4. (left) ^{15}N-HSQC spectrum of ApoL1-NTD with labeled chemical shift positions for all residues for which assignments were determined. (right) Overlay of ^{15}N-HSQC spectra of ApoL1-NTD, collected at pH 7.0, 6.0, 5.5, and 5.0 at 37°C. The peaks visible at pH 7.0 remain at largely the same chemical shift values at lower pH values, suggesting that the ground-state conformation of the NTD is similar at pH 7.0 as at pH 5.5 and 5.0, but that this ground-state conformation is more completely stabilized and/or less prone to self-association at the lower pH values.

Supplementary Figure 5

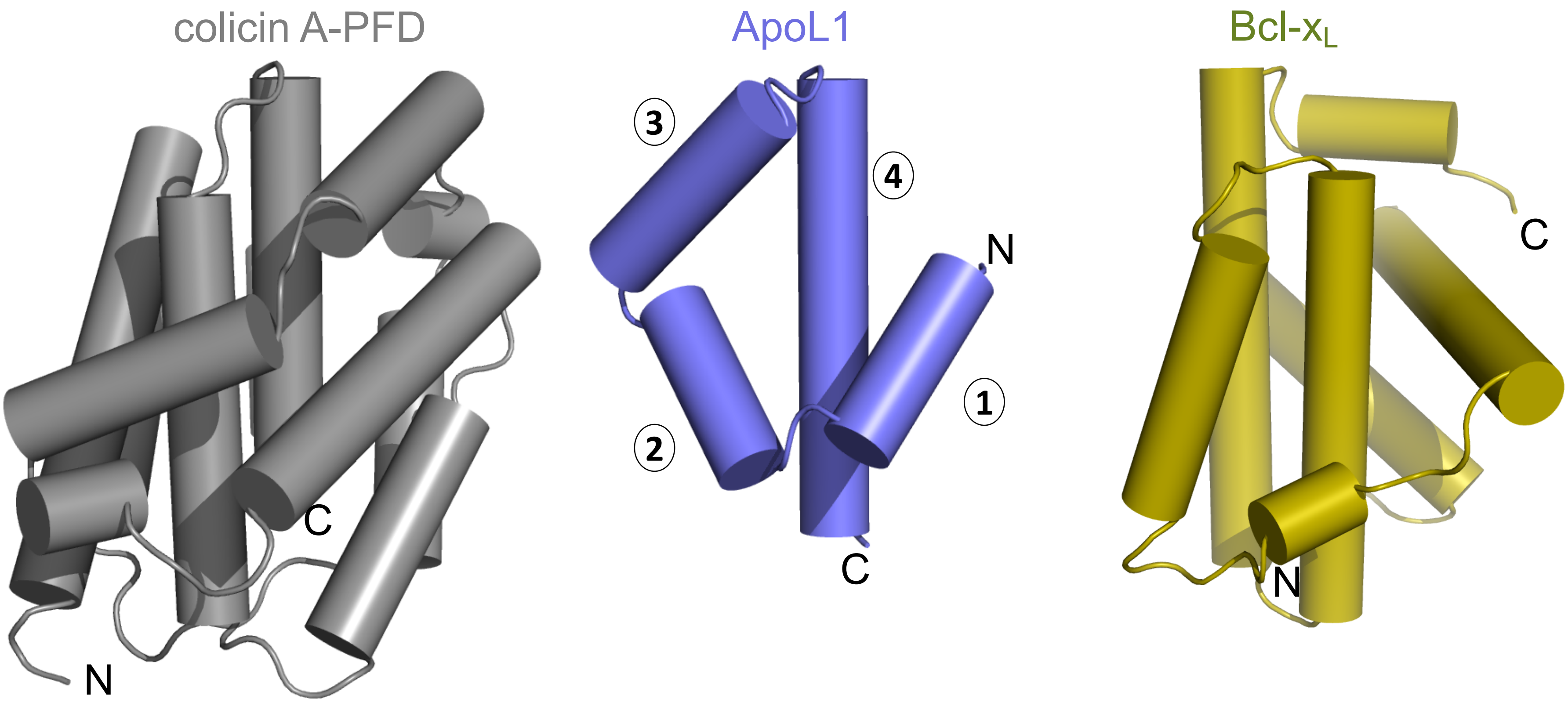

Supplementary Figure 5. The ApoL1-NTD fold is unlike either colicin-A-PFD or Bcl-x_L. Colicin-A-PFD (grey, from PDB 1COL), ApoL1-NTD (blue) and Bcl-x_L (yellow-green, from PDB 4QVE) are illustrated with α-helices as cylinders. The ApoL1-NTD shown is from the Fab3B6 complex, chain "C". Neither DALI nor PDBeFold identified structural correspondence in pairwise comparisons, consistent with the lack of overall similarity apparent here.

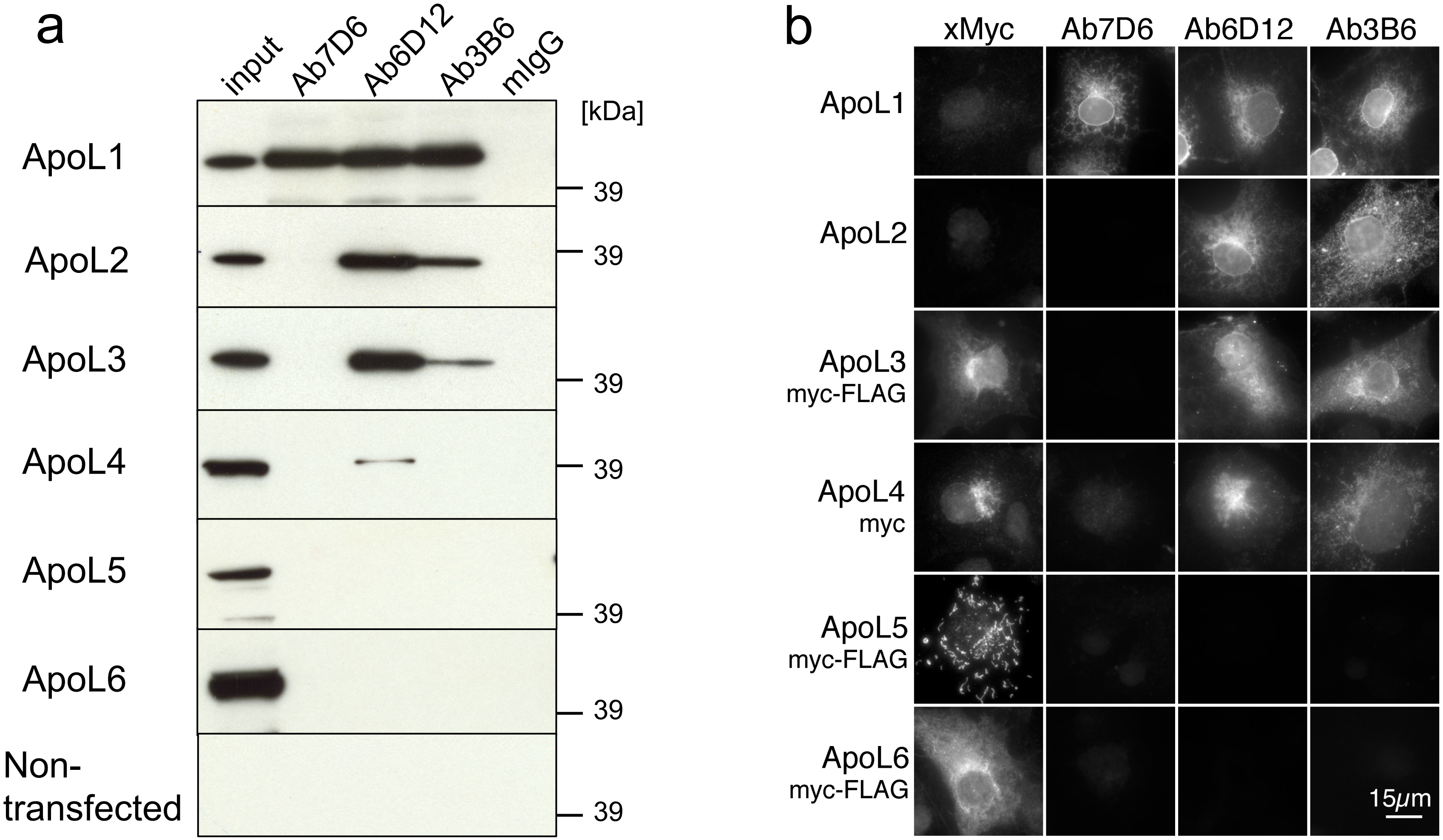

Supplementary Figure 6. Cross-reactivity of anti-ApoL1 antibodies with ApoL family members. COS-7 cells were transfected with untagged *ApoL1* or *ApoL2* or with C-terminally tagged *ApoL3-Myc-Flag*, *ApoL4-Myc*, *ApoL5-Myc-Flag*, or *ApoL6-Myc-Flag*. **a.** Immunoprecipitation of lysates of COS-7 cells incubated with the anti-ApoL1 antibodies Ab7D6, Ab6D12, Ab3B6 or mIgG control bound to magnetic beads. Eluted ApoL1-6 were detected by with Proteintech anti-ApoL1/2 antibody (for ApoL1 and ApoL2) or anti-Myc antibody (for ApoL3-6). The blot is representative of at least two independent experiments. **b.** Immunofluorescence of transiently transfected COS7 cells with anti-ApoL1 antibodies Ab7D6, Ab3B6 or Ab6D12 (+ Alexa488-anti mouse) along with rabbit anti-myc 71D10 (+ Dy649 anti-rabbit) to verify transfection for ApoL3-6 (column 1), using the PFA/Triton method. The staining is representative of at least two independent experiments. Ab3B6 only recognized the highest ApoL4-expressing cells by immunofluorescence (shown here), suggesting a very weak binding, in agreement with the lack of binding by immunoprecipitation.

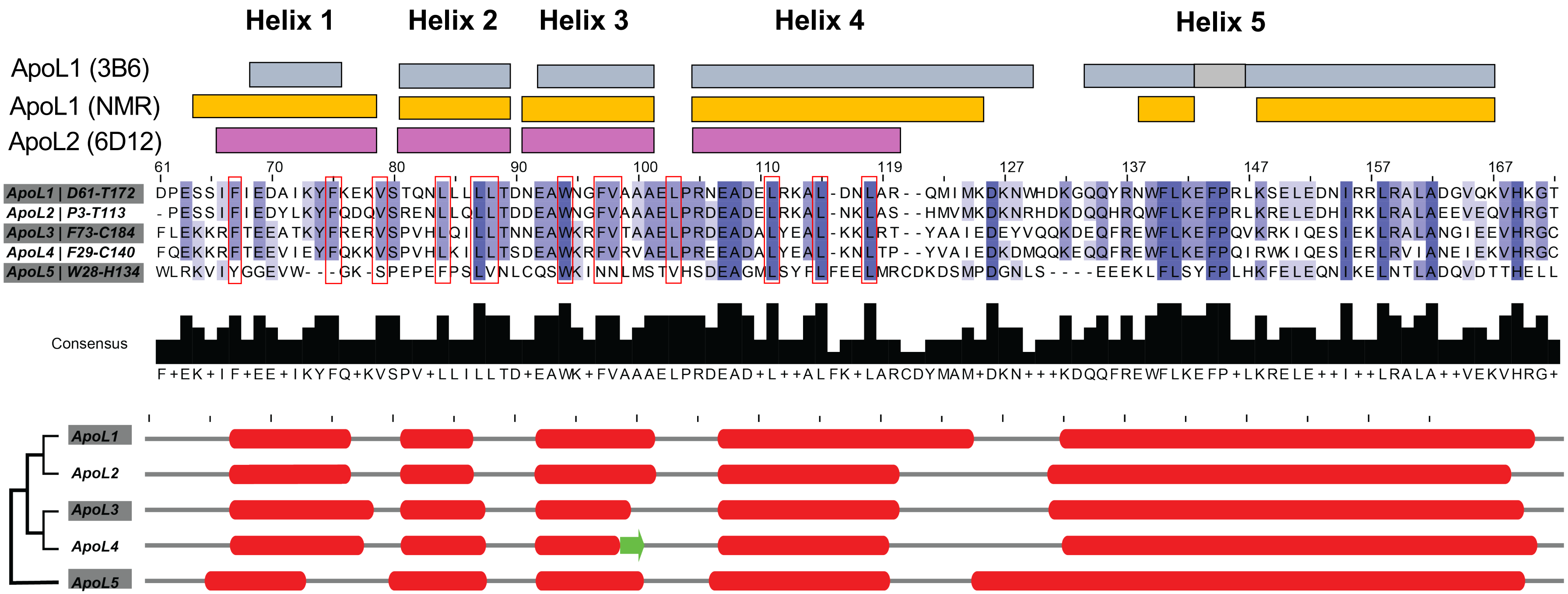

Supplementary Figure 7. Alignments and secondary structure predictions of the NTDs of ApoL1-5 in comparison to the experimentally determined helix boundaries of ApoL1 and ApoL2. ApoL6 is not shown, since it is missing helices 1-2 and is unable to form the four-helix conformation. The sequence alignment shows that the hydrophobic residues, which stabilize the four-helix arrangement, are conserved in ApoL1-4, but less in ApoL5 (red boxes). Secondary structure predictions (JPred4) (bottom; red bars) of ApoL1 and ApoL2 agree with the experimentally determined helix boundaries by crystallography (grey bars: Fab3B6:ApoL1-NTD complex, chain C; pink bars: Fab6D12:ApoL2-NTD complex) and by NMR (ApoL1-NTD, yellow bars). The predicted helical segments of ApoL3 and ApoL4 are in good agreement with those of ApoL1 and ApoL2.

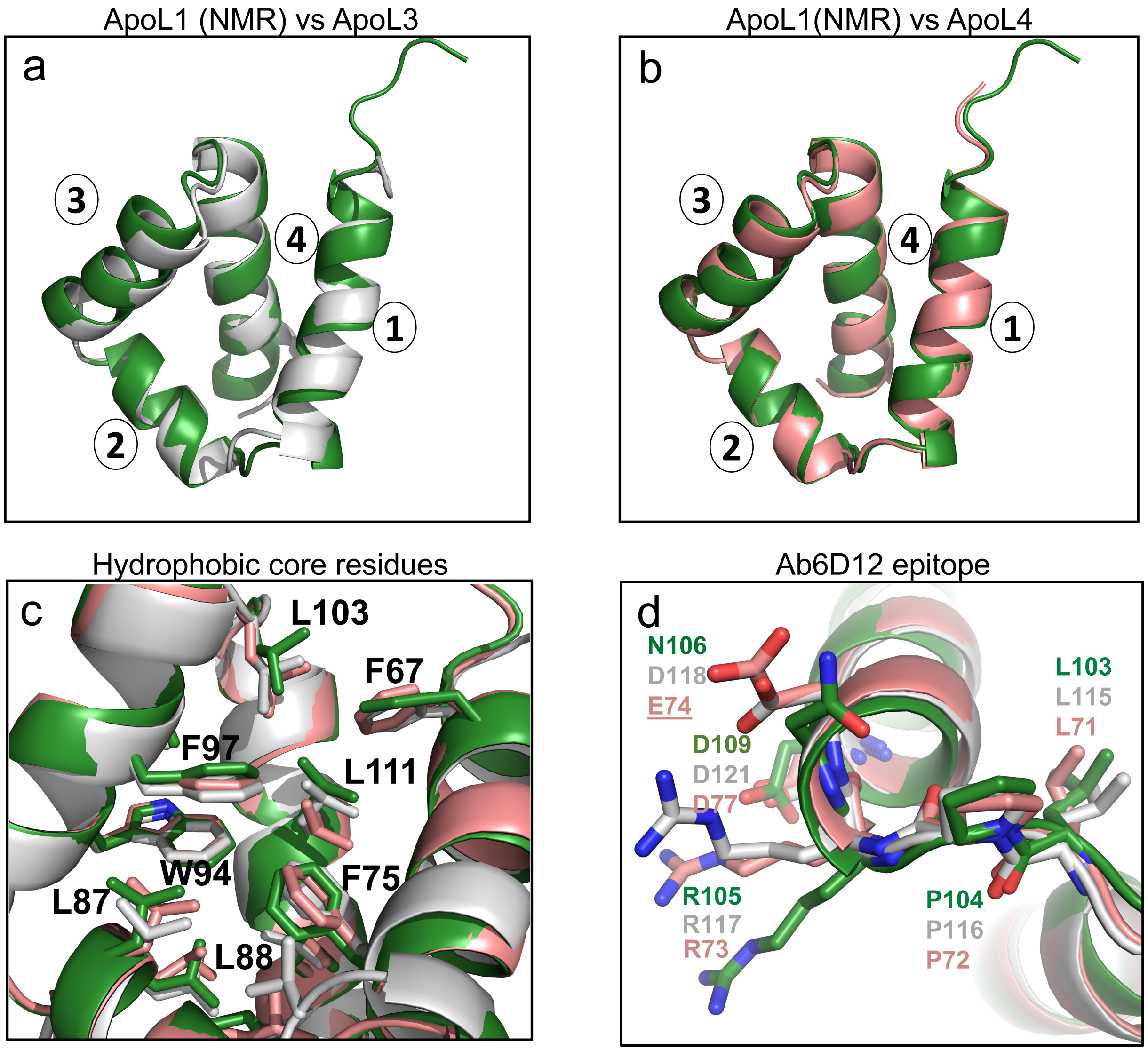

Supplementary Figure 8. Structural models of the four-helix core region of ApoL3 and ApoL4. The models were generated by the Rosetta prediction server using the NMR structure of ApoL1 as a template. **a.** Overlay of the ApoL3 model (grey cartoon) with helices 1-4 of the ApoL1 structure (green cartoon). **b.** Overlay of the ApoL4 model (pink cartoon) with helices 1-4 of the ApoL1 structure (green cartoon). **c.** Close-up of core-stabilizing hydrophobic residues (side chains as sticks) in an overlay of the ApoL1 structure (green cartoon) with the models of ApoL3 (grey cartoon) and ApoL4 (pink cartoon). The side chain numbers refer to ApoL1 (see Supplementary Fig. 7 for residue alignment). **d.** Close-up of the conserved Fab6D12 epitope in a superposition of all three structures (same color code as in A-C) with important contact residues as sticks, including the problematic E74 of ApoL4 (in pink, underlined).

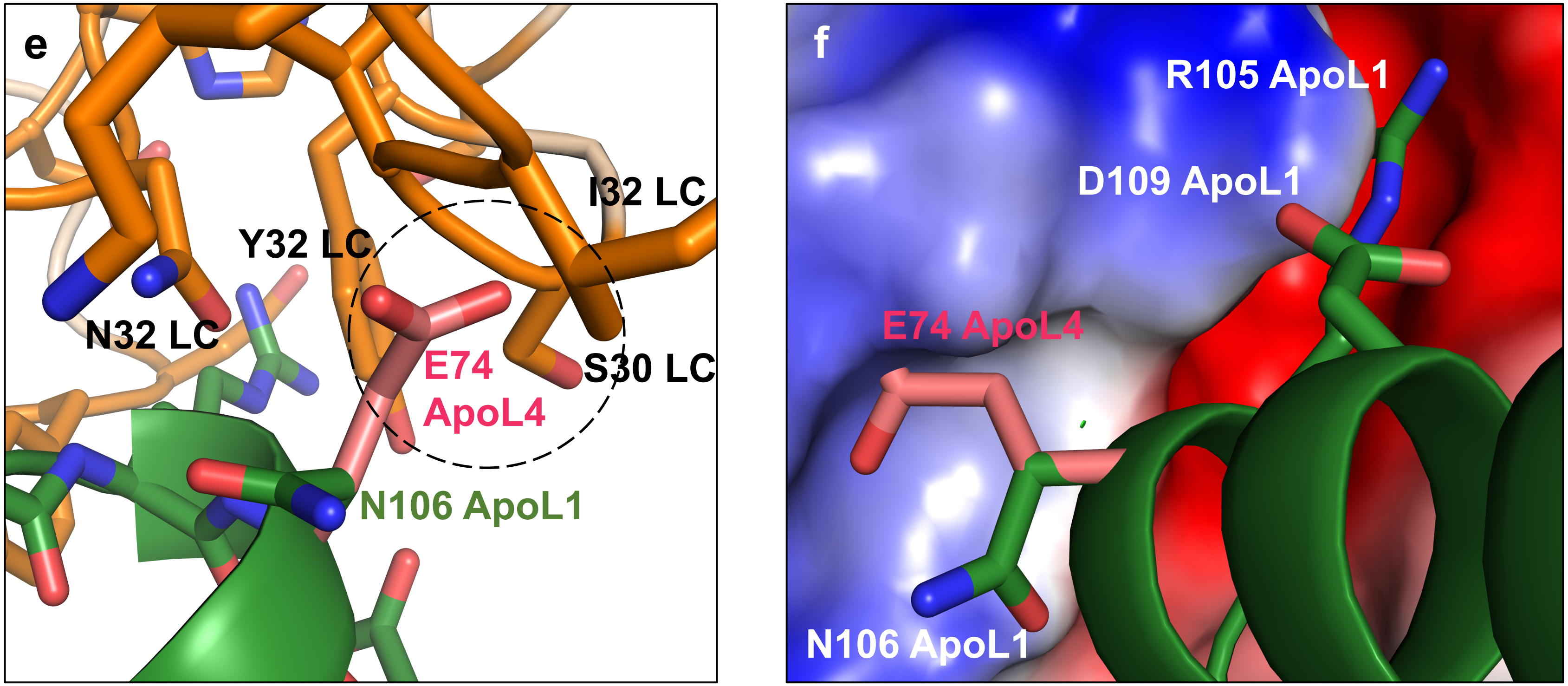

Supplementary Figure 8. e. Modelled ApoL4-E74 residue superimposed on the Fab6D12:ApoL1-NTD structure (ApoL1-NTD in green; Fab6D12 in orange). The ApoL4 residue E74 (pink) clashes with the Fab6D12 light chain residue Ser30, explaining the weak binding of Ab6D12 with ApoL4 in immunoprecipitation experiments (Supplementary Fig. 6A) **f.** Close-up of clash between ApoL4-E74 (as pink stick) with Fab6D12, which is shown in surface representation and colored according to approximate net electrostatic potential (blue, positive; red, negative).

Fab6D12 epitope

ApoL1	98 - 109	V	A	A	A	E	L	P	R	N	E	A	D
ApoL2	39 - 50	V	A	A	A	E	L	P	R	D	E	A	D
ApoL3	110 - 121	V	T	A	A	E	L	P	R	D	E	A	D
ApoL4	66 - 77	V	R	V	A	E	L	P	R	E	E	A	D
ApoL5	61 - 72	N	L	M	S	T	V	H	S	D	E	A	G

Fab7D6 epitope

ApoL1	152 - 168	E	D	N	I	R	R	L	R	A	L	A	D	G	V	Q	K	V
ApoL2	87 - 103	E	D	H	I	R	K	L	R	A	L	A	E	E	V	E	Q	V
ApoL3	164 - 180	Q	E	S	I	E	K	L	R	A	L	A	N	G	I	E	E	V
ApoL4	120 - 136	Q	E	S	I	E	R	L	R	V	I	A	N	E	I	E	K	V
ApoL5	114 - 130	E	Q	N	I	K	E	L	N	T	L	A	D	Q	V	D	T	T
ApoL6	54 - 70	K	G	N	I	D	K	L	R	A	L	A	D	D	I	D	K	T

Supplementary Figure 9. Sequence alignments of Fab6D12 and Fab7D6 epitopes. Conserved residues are boxed in pink color. The ApoL6 sequence is not shown in the upper panel, since ApoL6 is missing helices 1-2 and, therefore, will be unable to bind to bind to Fab6D12.

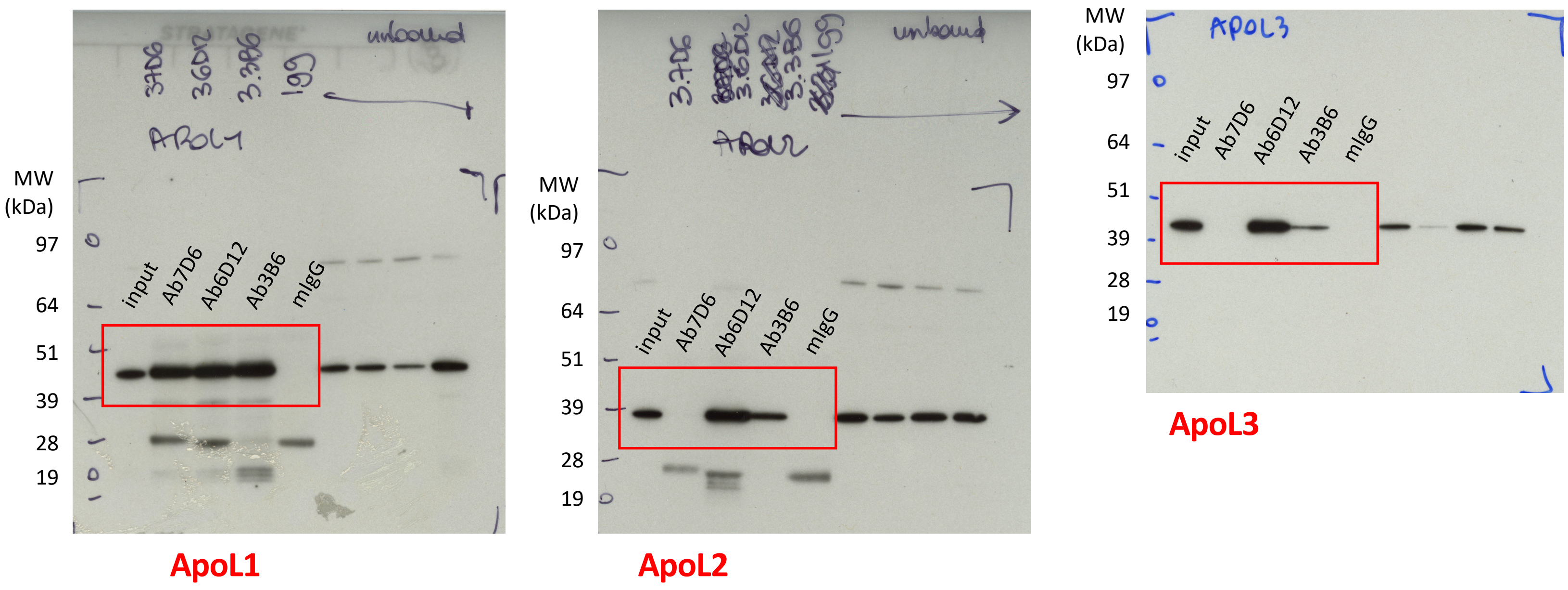

Supplementary Figure 10. Uncropped western blots related to Supplementary Figure 6.

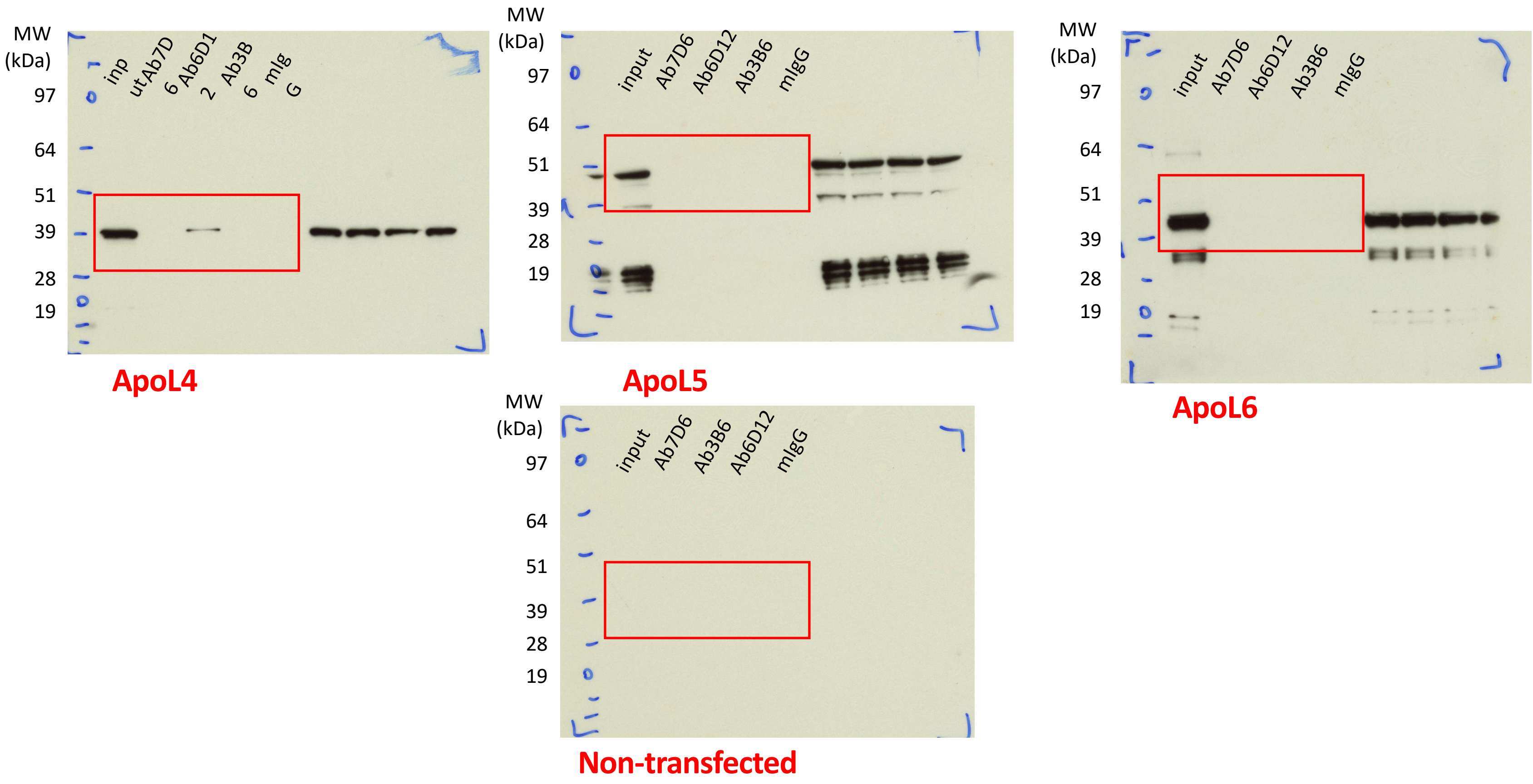

Supplementary Figure 11. Uncropped western blots related to Supplementary Figure 6.
