# Supplementary material for: Structures of the ApoL1 and ApoL2 N-terminal domains reveal a non-classical four-helix bundle motif
Source: Commun Biol. 2021 Jul 27;4:916. doi: 10.1038/s42003-021-02387-5 (PMC8316464; doi:10.1038/s42003-021-02387-5)
Supplement: Supplementary file 3 — Reporting Summary [file 42003_2021_2387_MOESM3_ESM.pdf]

nature research

Corresponding author(s): Daniel Kirchhofer

Last updated by author(s): Jun 2, 2021

# Reporting Summary

Nature Research wishes to improve the reproducibility of the work that we publish. This form provides structure for consistency and transparency in reporting. For further information on Nature Research policies, see our Editorial Policies and the Editorial Policy Checklist.

## Statistics

For all statistical analyses, confirm that the following items are present in the figure legend, table legend, main text, or Methods section.

| n/a | Confirmed | |
|---|---|---|
| ☐ | ☒ | The exact sample size ($n$) for each experimental group/condition, given as a discrete number and unit of measurement |
| ☐ | ☒ | A statement on whether measurements were taken from distinct samples or whether the same sample was measured repeatedly |
| ☒ | ☐ | The statistical test(s) used AND whether they are one- or two-sided<br>*Only common tests should be described solely by name; describe more complex techniques in the Methods section.* |
| ☒ | ☐ | A description of all covariates tested |
| ☒ | ☐ | A description of any assumptions or corrections, such as tests of normality and adjustment for multiple comparisons |
| ☒ | ☐ | A full description of the statistical parameters including central tendency (e.g. means) or other basic estimates (e.g. regression coefficient) AND variation (e.g. standard deviation) or associated estimates of uncertainty (e.g. confidence intervals) |
| ☒ | ☐ | For null hypothesis testing, the test statistic (e.g. $F$, $t$, $r$) with confidence intervals, effect sizes, degrees of freedom and $P$ value noted<br>*Give P values as exact values whenever suitable.* |
| ☒ | ☐ | For Bayesian analysis, information on the choice of priors and Markov chain Monte Carlo settings |
| ☒ | ☐ | For hierarchical and complex designs, identification of the appropriate level for tests and full reporting of outcomes |
| ☒ | ☐ | Estimates of effect sizes (e.g. Cohen's $d$, Pearson's $r$), indicating how they were calculated |

*Our web collection on statistics for biologists contains articles on many of the points above.*

## Software and code

Policy information about availability of computer code

| | |
|---|---|
| Data collection | For Xray data collection, refinement and graphics we used the following software versions: PHENIX (phenix.refine: 1.9_1692)<br>PyMOL v1.8.6.2 Enhanced for Mac OS X; BUSTER 2.11.6; CCP4 7.1 dials -v1-9-2; XDS 1.1.5; Phaser 2.5.5; Coot 0.8.6; refmac 5.8.0049, Aimless Version: 1.1.7; STARANISO OpenMP version: 1.10.9 |
| Data analysis | SPR data in Table 1 and Suppl. Table were analyzed by using the GE Biacore S200 instrument and the Biacore T200 evaluation software version 3.0, respectively.<br>For BLI experiments the data analysis was performed with Octet software (Octet Red System, PALL ForteBio)<br>For SEC-MALS experiments the data were analyzed using the ASTRA 7.2 software (Wyatt Technology)<br>Immunofluorescence Images (Suppl. Fig. 6B) were acquired on a Zeiss AxioImager M2 with a PhotoMetrics HQ2 camera, and processed in Adobe Photoshop CC2019. |

For manuscripts utilizing custom algorithms or software that are central to the research but not yet described in published literature, software must be made available to editors and reviewers. We strongly encourage code deposition in a community repository (e.g. GitHub). See the Nature Research guidelines for submitting code & software for further information.

## Data

Policy information about availability of data

All manuscripts must include a data availability statement. This statement should provide the following information, where applicable:

- Accession codes, unique identifiers, or web links for publicly available datasets
- A list of figures that have associated raw data
- A description of any restrictions on data availability

The X-ray structures of Fab6D12:ApoL1-NTD (PDB ID: 7LF7), Fab6D12:ApoL2-NTD (PDB ID: 7LF8), Fab3B6:ApoL1-NTD (PDB ID: 7LFA), Fab7D6:ApoL1-NTD (PDB ID: 7LFB) and Fab7D6:ApoL1-peptide (PDB ID: 7LFD), as well as the NMR structure of ApoL1-NTD (PDB ID: 7L6K) have been submitted to the Protein Data Bank. Reagents are available upon request.

# Field-specific reporting

Please select the one below that is the best fit for your research. If you are not sure, read the appropriate sections before making your selection.

☒ Life sciences ☐ Behavioural & social sciences ☐ Ecological, evolutionary & environmental sciences

For a reference copy of the document with all sections, see nature.com/documents/nr-reporting-summary-flat.pdf

# Life sciences study design

All studies must disclose on these points even when the disclosure is negative.

| | |
|---|---|
| Sample size | N/A to the crystal structures and NMR structure |
| Data exclusions | No data were excluded |
| Replication | N/A to the crystal structures and NMR structure (only done once).<br><br>SPR experiments for determining Kd values were carried out at least three times on different experimental days using freshly made reagents and newly made dilution series of analytes (Table 3 and Suppl. Table 1).<br><br>Molecular mass determinations by SEC-MALS were carried out three times on different experimental days to determine the average masses of the proteins and protein complexes ± standard deviation (Suppl. Fig.1A).<br><br>The biolayer interferometry result shown in Suppl. Fig.1B was reliably reproduced by three experiments performed at different days with newly made reagents each time.<br><br>Immunoprecipitation and immunofluorescence results depicted in Suppl. Fig. 6A and 6B were reliably reproduced by at least two experiments performed at different days and with newly made reagents each time. Transient transfections at different experimental days were done with the same maxi prep except for ApoL1 and ApoL2 where two or more maxi preps were used. |
| Randomization | N/A |
| Blinding | N/A |

# Reporting for specific materials, systems and methods

We require information from authors about some types of materials, experimental systems and methods used in many studies. Here, indicate whether each material, system or method listed is relevant to your study. If you are not sure if a list item applies to your research, read the appropriate section before selecting a response.

## Materials & experimental systems

| n/a | Involved in the study | |
|---|---|---|
| ☐ | ☒ | Antibodies |
| ☐ | ☒ | Eukaryotic cell lines |
| ☒ | ☐ | Palaeontology and archaeology |
| ☒ | ☐ | Animals and other organisms |
| ☒ | ☐ | Human research participants |
| ☒ | ☐ | Clinical data |
| ☒ | ☐ | Dual use research of concern |

## Methods

| n/a | Involved in the study | |
|---|---|---|
| ☒ | ☐ | ChIP-seq |
| ☒ | ☐ | Flow cytometry |
| ☒ | ☐ | MRI-based neuroimaging |

## Antibodies

| | |
|---|---|
| Antibodies used | The Ab6D12, Ab3B6 and Ab7D6 have been published (refs#61 and #64) and have been validated by SPR (Suppl. Table 1, method section) and by FACS using different cell lines and HDL particles (refs#61 and #64).<br><br>For SPR experiments reported in Suppl. Table 1 the capture antibody was anti-murine Fc antibody (Cytiva, cat# BR100838).<br><br>Immunoprecipitation experiments in Suppl. Fig. 6A: ApoL1 and ApoL2 on nitrocellulose membranes were detected by anti-ApoL1/2 rabbit polyclonal antibody (Proteintech 11486-2-AP) and Myc-tagged ApoL3-6 were detected with rabbit monoclonal anti-Myc tag 71D10 (Cell Signaling, 2278S) followed by HRP-anti-rabbit IgG (Jackson 711-036-152).<br><br>Immunofluorescence experiments in Suppl. Fig. 6B: The murine antibodies Ab6D12, Ab3B3 and Ab7D6 were detected by Alexa488-anti-mouse (Jackson ImmunoResearch 715-546-150). ApoLs were co-stained with anti-myc tag rabmab 71D10 (Cell Signaling, 2278S) and Dy649-anti-rabbit (Jackson ImmunoResearch 712-496-152). |
| Validation | anti-murine Fc antibody (Cytiva, cat# BR100838) description and validation: https://cdn.cytivalifesciences.com/dmm3bwsv3/AssetStream.aspx?mediaformatid=10061&destinationid=10016&assetid=16851<br>anti-ApoL1/2 rabbit polyclonal antibody (Proteintech 11486-2-AP) has been validated by siRNA experiment and is documented by numerous publications: https://www.ptglab.com/products/APOL1-Antibody-11486-2-AP.htmassetid=16851<br>rabbit monoclonal anti-Myc tag 71D10 (Cell Signaling, 2278S) has been validated by cell transfection experiment using Myc-Bcl2: https://www.cellsignal.com/products/primary-antibodies/myc-tag-71d10-rabbit-mab/2278<br>HRP-anti-rabbit IgG (Jackson 711-036-152) has been validated for species IgG specificity using ELISA as described: https://www.jacksonimmuno.com/catalog/products/711-036-152.<br>Alexa488-anti-mouse (Jackson ImmunoResearch 715-546-150) has been validated for species IgG specificity using ELISA as described:https://www.jacksonimmuno.com/catalog/products/715-546-150<br>The HRP-anti-rabbit IgG (Jackson 711-496-152) has a new cat. number: 711-606-152: According to the manufacturer (https://www.dianova.com/en/shop/711-606-152-donkey-fab2-anti-rabbit-igg-hl-alexa-fluor-647-minx-bockgogphshohumsrtsh/), the antibody has been tested by ELISA and/or solid-phase adsorbed to ensure minimal cross-reaction with bovine, chicken, goat, guinea pig, Syrian hamster, horse, human, mouse, rat, and sheep serum proteins, but it may cross-react with immunoglobulins from other species. |

## Eukaryotic cell lines

Policy information about cell lines

| | |
|---|---|
| Cell line source(s) | COS7 cells (African green monkey kidney) were obtained from the Genentech cell culture facility (initially purchased from ATCC) |
| Authentication | STR (Short Tandem Repeat) profiling was used to authenticate the COS7 cell line by the Genentech cell culture facility |
| Mycoplasma contamination | COS7 cells were mycoplasma tested |
| Commonly misidentified lines<br>(See ICLAC register) | COS7 is not a misidentified cell line |
